# Role of miR-223 in the pathophysiology of liver diseases

**DOI:** 10.1038/s12276-018-0153-7

**Published:** 2018-09-26

**Authors:** Dan Ye, Tianbao Zhang, Guohua Lou, Yanning Liu

**Affiliations:** 1 Department of Endocrinology and Metabolism, The First Affiliated Hospital of School of Medicine, Zhejiang University, Hangzhou, China; 2 State Key Laboratory for Diagnosis and Treatment of Infectious Diseases, Collaborative Innovation Center for Diagnosis and Treatment of Infectious Diseases, The First Affiliated Hospital of School of Medicine, Zhejiang University, Hangzhou, China

## Abstract

MiRNAs are small, noncoding RNAs, which can regulate gene expression posttranscriptionally, and they have emerged as key factors in disease biology by aiding in disease development and progression. MiR-223 is highly conserved during evolution and it was first described as a modulator of hematopoietic lineage differentiation. MiR-223 has an essential part in inflammation by targeting the nuclear factor-κB pathway and the nucleotide-binding oligomerization domain-like receptor protein 3 inflammasome. Recent studies have shown that miR-223 expression is deregulated in various types of liver diseases, including hepatitis virus infections, alcohol-induced liver injury, drug-induced liver injury, non-alcoholic fatty liver disease, cirrhosis, and hepatocellular carcinoma. As inflammatory and immune factors are involved in the occurrence and progress of liver diseases, deregulated miR-223 may participate in the pathogenesis of these conditions by influencing neutrophil infiltration, macrophage polarization, and inflammasome activation. This review first summarizes the present understanding of the biological functions of miR-223, including its gene location and transcription regulation, as well as its physiological role in hematopoietic differentiation. This review then focuses on the role of miR-223 in liver pathophysiology and its potential applications as a diagnostic biomarker and therapeutic target in liver diseases.

## Liver disease: a tiny trigger for liver damage

A tiny RNA molecule involved with gene regulation may offer an appealing target for diagnosing and treating various liver diseases. MicroRNA-223 (miR-223) was first identified as controlling gene activity in a wide variety of immune cells. A review from researchers led by Yanning Liu at China’s Zhejiang University in Hangzhou details how abnormal miR-223 also contributes to liver damage in a variety of conditions, although questions still remain about how it functions in different liver disorders. The authors highlight studies linking miR-223 with the development of fibrosis and cirrhosis, and with the inflammatory response to injury from drugs, alcohol, or infection. This could make this microRNA a useful diagnostic biomarker. The authors also identify therapeutic opportunities to modulate this molecule, referring to several studies on the manipulation of miR-223 to treat hepatitis.

## Introduction

MicroRNAs (miRNAs) are a class of highly conserved, chemically stable, small noncoding endogenous RNA (∼22 nt) molecules. Since their discovery in 1993^1^, miRNAs have been extensively studied due to their role in RNA-induced gene silencing at the posttranscriptional level. Approximately 17,000 miRNAs exist in more than 140 species^2^. MiRNA biogenesis is a complex process that begins in the cell nucleus where miRNAs are encoded as short inverted repeats with a double-stranded RNA stem loop. A long primary transcript (pri-miRNA) is transcribed by RNA polymerase II and may contain one or more hairpins. Before transport into the cytoplasm, hairpins are excised from pri-miRNAs by the double-strand-specific ribonuclease, Drosha-DGCR8 complex, to form precursor miRNAs (pre-miRNAs). Pre-miRNAs are then exported to the cytoplasm through Exportin-5 and Ran-GTP. In the cytoplasm, another protein complex containing the RNAseIII enzyme Dicer further processes pre-miRNAs into mature miRNAs, which are loaded into the RNA-induced silencing complex and then downregulate specific target mRNAs either by decreasing the transcript levels or by translational repression^3^. At present, 1881 precursors and 2588 mature human miRNAs are registered in the miRbase ver. 21.0. These miRNAs have been shown to control the expression of at least 60% of human protein-coding genes^4,5^. MiRNAs have important regulatory roles in modulating normal physiology, including development, tissue remodeling, metabolism, immunity, cell proliferation, cell differentiation, and intracellular signaling^6^, whereas dysregulated miRNAs are correlated with a variety of human diseases^7^.

Studies have demonstrated that miRNA expression signatures are highly tissue- and disease-specific, and approximately 70% of miRNAs in the whole body have been discovered in liver tissues^8^. By developing a tissue-specific mature miRNA deletion mouse model, in which Dicer1 function is absent in hepatoblast-derived cells, researchers have shed light on the liver-specific roles of miRNAs^9^. Despite the loss of mature miRNAs, hepatic basal functionality is maintained in these mice. However, mutant mice at 2–4 months of age exhibit progressive hepatocyte damage with elevated serum alanine aminotransferase and aspartate aminotransferase combined with significant hepatic steatosis, glycogen depletion, increased hepatocyte apoptosis, increased hepatocyte proliferation, and portal inflammation^9^. Interestingly, a different Dicer1-knockout mouse model, in which Dicer1 is conditionally deleted in mature hepatocytes, fetal stage-specific genes are persistently expressed, resulting in increased hepatocyte proliferation and apoptosis, as well as spontaneous development of hepatocellular carcinoma (HCC)^10^. These data demonstrate that miRNAs have critical roles in hepatic physiology and pathology.

**Fig. 1:**
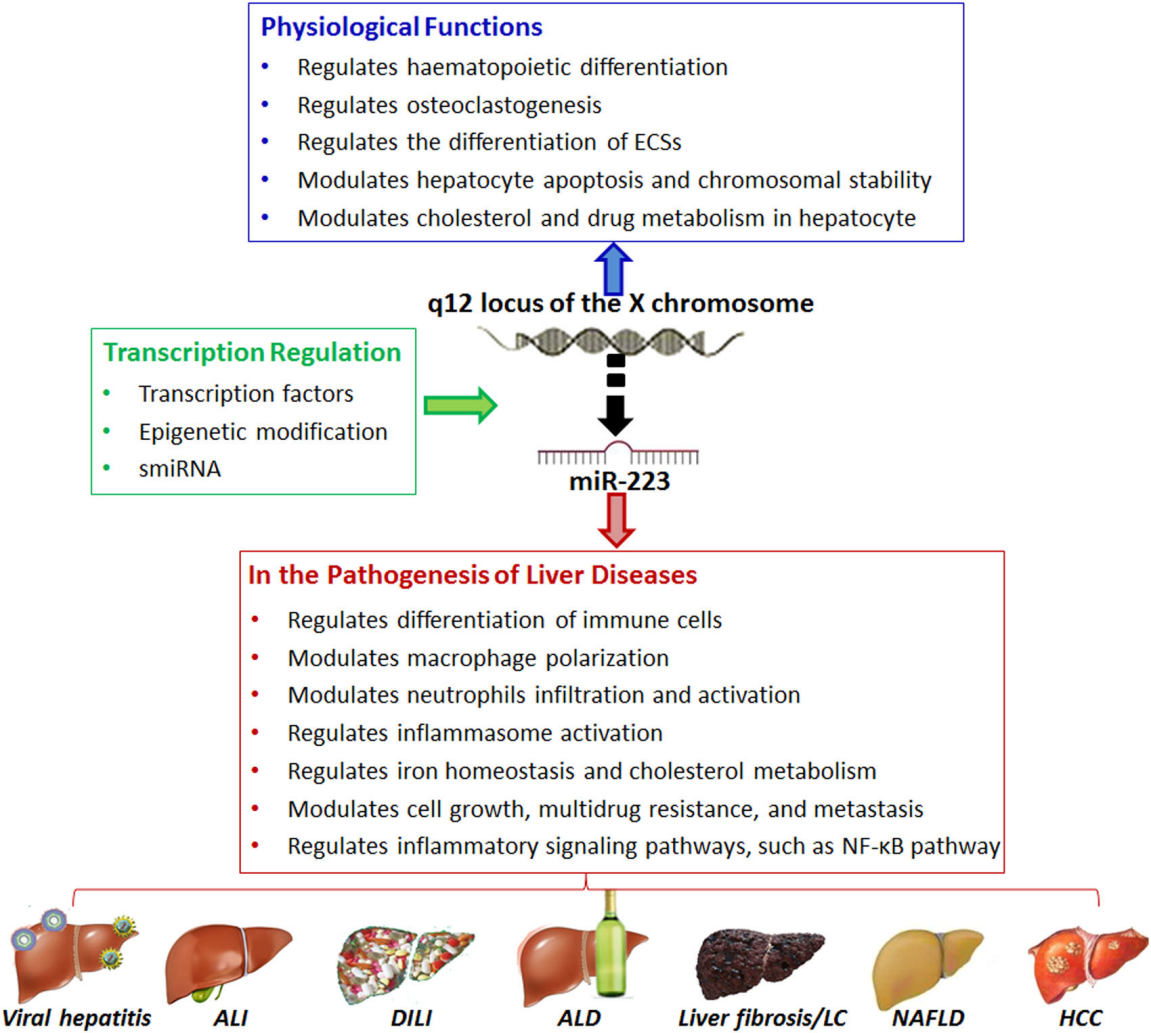
MiR-223 has important roles in normal physiology and hepatic pathogenesis. MiR-223 is located within the q12 locus of the X chromosome and is regulated by several transcription factors, epigenetic modification, and smiR-223. MiR-223 has important roles in physiological condition, including hematopoietic differentiation, osteoclastogenesis, embryonic stem cell (ESC) differentiation, cell apoptosis, chromosomal stability, hepatic cholesterol metabolism, and hepatic drug metabolism. MiR-223 is also involved in the pathogenesis of various liver diseases by influencing immune cell differentiation, neutrophil infiltration, macrophage polarization, inflammasome activation, iron homeostasis, metabolic signaling pathways, and inflammatory signaling pathways

Many clinical studies have shown that miRNA deregulation may be a key pathogenic factor in many liver diseases, including viral hepatitis, liver fibrosis, drug-induced liver injury (DILI), metabolic lever diseases, acute liver diseases, and HCC^11,12^. MiR-122, one of the most abundant adult hepatic miRNAs, regulates a wide range of key gene networks, such as hepatic circadian rhythm, lipid metabolism, and cell differentiation^13^. Recently, increasing evidence has suggested that miR-223 is one of the key factors in the development and homeostasis of the immune system^14^, and may also have an essential part in both inflammation disorders and various liver diseases. The present article reviews current knowledge on the physiopathology of miR-223 starting with its biogenesis and biological functions, and it focuses on the emerging role of miR-223 in liver pathology and its potential for diagnosis and treatment in liver diseases (Fig. 1).

### MIR-223 biological functions

#### MiR-223 gene location and transcription regulation

The miR-223 encoding gene is located within the q12 locus of the X chromosome^15^. MiR-223 sequence is highly conserved during evolution, suggesting its potential role in essential physiological events. MiRNA-223 was first identified in the hematopoietic system^16^. As the miR-223 gene resembles a “myeloid gene,” its expression is regulated by several myeloid transcription factors, including PU.1, CAAT/enhancer-binding proteins (C/EBP)-α and -β, and nuclear factor I-A (NFI-A). PU.1 binds to two separate sites within the miR-223 promoter to activate miRNA expression, which in turn induces osteoclastogenesis^17,18^. Similarly, C/EBP-α binds to the miR-223 promoter and increases its expression, which undergoes a specific activation to promote granulocyte differentiation^19^. C/EBP-α has also been shown to strongly enhance miR-223 promoter activity by combination with PU.1^20^. However, NFI-A maintains low levels of miR-223, as it competes for binding with C/EBP-α, thus decreasing granulocyte and osteoclast differentiation^18,19^. Interestingly, both NFI-A and C/EBP-β act as direct targets of miR-223, thus forming negative feedback loops between the target genes and their corresponding miRNA^19,21^. Hence, miR-223 expression is coordinately affected by the combined actions of different myeloid transcription factors. The dysregulation of transcription factors may also contribute to the aberrant expression of miR-223 in multiple types of cancer, inflammatory diseases, and other pathological processes^14,22^.

Recently, increasing evidence has suggested that epigenetic mechanisms, such as DNA methylation and posttranslational modifications of nucleosomal histone proteins, are also involved in miRNA regulation. Heterochromatic silencing of miR-223 can be induced by recruitment of chromatin-remodeling enzymes on the pre-miR-223 promoter^23^. The prevention of acetylated histone H3 and C/EBP-α recruitment to the miR-223 promoter also reduces miR-223 expression^24^.

A pioneer study has demonstrated that the existence of semi-miRNAs (smiRNAs), as 12 nt-long RNA species corresponding to the 5′-region of mature miRNAs, may participate in regulating miRNA activity in vivo^25^. In the investigation of the existence and role in mRNA regulation of shorter RNA species derived from miRNAs, researchers serendipitously discovered a significant number of smiRNA sequences, derived from their corresponding pre-miRNA species processed by a Dicer complex, and can act as natural miRNA anti-sense RNAs to neutralize the function of miRNA species of sufficient base pair complementarity. By using smiR-223 derived from pre-miR-223 as a model, researchers further showed that although smiR-223 cannot directly regulate mRNA activity through mRNA translational repression, it can interfere with the ability of miR-223 to mediate gene silencing either through mRNA cleavage or mRNA translational repression^25^. This condition implies the ability of miR-223 to provide an additional layer of control over miR-223 activity.

### Physiological function

As miR-223 is preferentially expressed in the hematopoietic system, it is considered to have important roles in hematopoietic differentiation by affecting hematopoietic stem cells, myeloid cells, erythroid cells, and lymphoid cells at various stages of their development^16^.

The first important role of miR-223 was discovered in granulopoiesis according to its highest expression level in granulocytes^26,27^. MiR-223 overexpression is sufficient to induce myeloid precursor cell differentiation, whereas miR-223 inhibition reduces the efficiency of RA-induced differentiation^19^. A study using the miR-223^-/Y^ mouse model showed that miR-223 is not strictly essential for granulocyte differentiation but is required for normal maturation of granulocytes and regulation of granulocyte compartment size. Marked neutrophilia and hyperplasia of the bone marrow granulocyte compartment were observed in this model^16^. This finding was validated by bone marrow reconstitution with hematopoietic stem cells stably overexpressing miR-223 sponge sequence to phenocopy the miR-223^-/Y^ mouse ^28^.

In addition to granulocyte differentiation, miR-223 has a role in monocyte/macrophage differentiation by repressing its target, IκB kinase subunit-α (IKK-α). In macrophage differentiation, repressed miR-223 expression induces IKK-α expression followed by the repression of nuclear factor-κB (NF-κB) pathways^29^. By using an antagomiR inhibitor of miR-223, the granulocyte-macrophage colony-stimulating factor-induced differentiation of monocytes and phorbol-12-myristate-13-acetate (PMA)-induced differentiation of THP1 cells can be abolished, further demonstrating the important role of miR-223 for myeloid differentiation^30^. Interestingly, miR-223-rich microvesicles (exosomes or microparticles) derived from differentiated macrophages sufficiently induce differentiation in recipient monocytes^31^. Unlike in monocytes, however, several studies have shown that miR-223 is downregulated in macrophages^29,32^. As miR-223 inhibition implies a strong effect on macrophage differentiation, a low functional level of this miRNA may be sufficient for monocyte differentiation.

In addition, miR-223 is involved in the regulation of erythroid and megakaryocyte differentiation by targeting LIM domain only 2 (LMO2)^22,33^. In K562 cells, an erythroid–megakaryocyte cell line, miR-223 expression is downregulated during haemin-induced erythroid differentiation but upregulated during PMA-induced megakaryocytic differentiation^32^. MiR-223 can also target and regulate the expression of the LMO2 transcription factor, a bridging molecule that assembles an erythroid DNA-binding complex, including the GATA-binding factor-1^33^. During erythroid differentiation, miR-223 downregulation increases LMO2 level and then promotes the differentiation of K562 cells into erythrocytes.

MiR-223 also has an important role in osteoclast formation and the regulation of bone remodeling as it is expressed in osteoclast precursors. Knockdown of miR-223 decreases the receptor activator of NFκB ligand (RANKL)-induced formation of osteoclast-like cells, whereas overexpression of miR-223 exerts a similar effect, suggesting that an appropriate level of miR-223 expression is important for normal osteoclastogenesis^34^. Interestingly, knockdown of miR-223 results in an increased production of the target gene, NFI-A, a suppressor of osteoclastogenesis. Bioinformatics suggests that the 3′-untranslated region (UTR) of NFI-A may have a potential miR-223-binding site. Thus, in osteoclast differentiation, miR-223 potentially targets NFI-A to induce expression of macrophage colony-stimulating factor receptor, which would modulate osteoclast differentiation, function, and survival^35^.

Recently, miR-223 has been demonstrated to regulate human embryonic stem cell (hESC) differentiation by targeting the insulin-like growth factor-1 receptor (IGF-1R)/Akt signaling pathway^36^. Based on bioinformatics prediction of complementary binding sites in the 3′-UTR of IGF-1R mRNA, miR-223 is assumed as a direct regulator of IGF-1R, which can modulate hESC development by regulating its downstream kinase, Akt. Moreover, miR-223 inhibition maintains the undifferentiated state of hESCs, whereas addition of exogenous miR-223 induces hESC differentiation.

MiR-223 can also modulate the physiological function of parenchyma cells. In hepatocytes, miR-223 modulates cholesterol metabolism, drug metabolism, and cell apoptosis^37–40^. MiR-223 represses selective high-density lipoprotein cholesterol (HDL-C) uptake by inhibition of hepatic scavenger receptor class B type I (SR-BI), a high-affinity HDL receptor, by directly targeting its 3′-UTR^37^. Given that selective HDL-C uptake is a pivotal step of reverse cholesterol transport, miR-223 may modulate hepatic cholesterol metabolism. MiR-223 has also been shown to directly modulate the endogenous protein level and mRNA stability of cytochrome b5 in human hepatocytes^38^. Due to the role of b5 in cytochrome P450-catalyzed drug metabolism, the inter-individual variability of miR-223 expression may affect the inter- and intra-individual variability of drug pharmacokinetics. A decreased miR-223 level in HCC development indicates the role of miR-223 in maintaining normal hepatic physiological function. Reduced expression of miR-223 in hepatocytes has been verified to potentially predispose HCC development via dysregulated expression of STMN1, which is a downstream target of miR-223 and is considered as a pro-tumorigenic gene to induce chromosomal instability^39^. MiR-223 also regulates Fas-induced hepatocyte apoptosis and liver injury by targeting the IGF-1R signaling pathway^40^.

In summary, miR-223 is located within the X chromosome and is regulated by several transcription factors, epigenetic modification, and smiR-223. MiR-223 has important roles in hematopoietic differentiation. MiR-223 expression increases as granulocyte–monocyte progenitors differentiate into granulocytes but decreases during differentiation into monocytes, and miR-223 expression is further altered as these cells are activated or polarized. MiR-223 also regulates the differentiation of erythroids, megakaryocytes, osteoclasts, and ESCs. Finally, miR-223 is involved in modulating hepatic physiological function by affecting cholesterol and drug metabolism, as well as apoptosis and chromosomal stability in hepatocytes.

### MIR-223 in the pathogenesis of liver diseases

Aside from its involvement in the differentiation of various immune cells, miR-223 also influences activation patterns. For instance, miR-223 modulates macrophage polarization, inflammasomes, and NF-κB signaling^32,41^. By targeting Pknox1, an essential regulator for macrophage polarization, miR-223 steers classically activated pro-inflammatory phenotype (M1) of macrophages toward the alternative anti-inflammatory phenotype (M2)^41^. MiR-223 may also regulate the nucleotide-binding oligomerization domain-like receptor (NLR) inflammasome by targeting the NLR protein 3 (NLRP3) 3′-UTR^42^. Upon stimulation by a Toll-like receptor (TLR) ligand, many cell types need the NLRP3 inflammasome to initiate inflammatory responses and induce interleukin (IL)-1β production. The results have shown that overexpression of miR-223 prevents accumulation of NLRP3 protein and inhibits IL-1β production from the inflammasome. Furthermore, miR-223 may prevent macrophage hyperactivation by cooperating with other miRNAs to modulate the noncanonical NF-κB transcription factor pathway by regulating IKK-α expression^29^.

MiR-223 expression is deregulated in many types of liver diseases, including viral hepatitis, alcohol-induced liver injury, DILI, non-alcoholic fatty liver disease (NAFLD), cirrhosis and HCC (Table 1). Several studies have shown that inflammatory and immune factors are involved in the occurrence and progress of various liver diseases. Thus, it can be speculated that miR-223 influences macrophage polarization and inflammasome activation in pathological conditions.

**Table 1:** MiR-223 expression and function in liver diseases

Liver disease	miR-223 expression		Target	Clinical usage/function	Reference
	Circulating	Cellular/hepatic			
**Viral hepatitis**					
Chronic hepatitis C	↑	–	–	Correlated with SVR in CHC patients by IFN-based combination therapy	^45^
Chronic hepatitis C	–	↓	–	May be linked to chronic liver inflammation by regulating NF-κB pathway	^46^
Chronic hepatitis B	↑	–	–	Served as a novel biomarker for liver injury	^47^
**ALI**					
Mouse/rat model of I/R-induced ALI	↑	↑	ACSL3, EFNA1, RhoB	Represent a potential markers of I/R injury; Correlated with the concentrations of serum ALT and AST	^51^^–^^53^
Fas-Induced ALI in miR-223 KO mice	–	↓	IGF-1R	MiR-223 deficiency protects against Fas-induced hepatocyte apoptosis and liver Injury	^40^
Mouse model of Con A-induced ALI	–	↓	–	Suppress Kupffer cells activation via indirectly inhibitiing AIM2 pathway	^54^
**DILI**					
Mouse model of APAP-induced DILI	–	↑	IKKα	Terminate acute neutrophilic response via a mtDNA/TLR9/NF-κB/miR-223 negative feedback loop	^56^
**ALD**					
Chronic-plus-binge ethanol feeding mouse model	↑	↑	IL-6	Ameliorates alcoholic liver injury by inhibiting the IL-6-p47phox-oxidative stress pathway	^60^
**NAFLD**					
NAFLD/NASH cellular model	↓	↓	–	May be associated with the onset and progression of NAFLD and NASH	^64^
Mouse model of CFD diet caused NAFLD	–	↑	IRP1	Monitors hepatic iron metabolism	^65^
Apoe^−/−^ mouse model of CBD-induced hypercholesterolemic	–	↑	SR-BI, HMGCS1, SC4MOL, Sp3	Coordinates cholesterol homeostasis	^66^
**Cirrhosis/liver fibrosis**					
HCV-related cirrhosis	↓	–	–	Served as a biomarker for HCV-positive cirrhosis	^71^
HCV-related liver fibrosis	↑	–	–	Served as a non-invasive biomarker for fibrosis staging in HCV-related liver fibrosis	^72^
HBV-related liver fibrosis	↓	–	–	Served as a non-invasive biomarker for early diagnosis of HBV-related liver fibrosis	^73^
Mouse model of CCl_4_ or BDL-induced liver fibrosis	–	↑	–	Correlated with degree of liver injury and hepatic cell death	^52^
**HCC**					
HCC cell lines	–	↓	Rab1, ABCB1	Suppress cell growth and promote apoptosis in HCC cell lines via Rab1-mediated mTOR activation; increase HCC cell sensitivity to anticancer drugs via targeting ABCB1-mediated multidrug resistance	^76^^,^^78^
Orthotopically implanted mouse model of HCC	–	↓	Integrin αV	Inhibits HCC metastasis and negatively affects integrin αV-mediated cell migration	^24^
HCC patients	↓	↓	Stathmin1	Associated with the development of HCC; served as a non-invasive biomarker for HCC and its recurrence following OLT	^39^^,^^79^^,^^83^
Alcohol consumption in HBV-related HCC	–	↑	Fbxw7	Enhance the risk of alcohol-associated carcinogenesis of HCC	^77^
HBV-positive HCC	↓	↓	c-myc	Involved in the hepatocarcinogenesis and served as a non-invasive biomarker for HCC	^49^^,^^80^
HCV- related HCC	↓	–	–	Served as an early diagnostic biomarker of HCV-positive HCC	^71^^,^^81^

*ABCB1* ATP-binding cassette subfamily member 1, *ACSL3* acyl-CoA synthetase long-chain family member 3, *ALD* alcoholic liver disease, *ALI* acute liver injury, *CFD* choline- and folate-deficient, *DILI* drug-induced liver injury, *EFNA1* ephrin A1, *HFD* high-fat diet, *HMGCS1* sterol enzymes 3-hydroxy-3-methylglutaryl-CoA synthase 1, *IFN* interferon, *IGF-1R* insulin-like growth factor-1 receptor, *IKKα* IκB kinase α, *I/R* ischemia/reperfusion, *IRP1* iron regulatory protein 1, *OLT* orthotopic liver transplantation, *RhoB* ras homolog gene family member B, *SR-BI* scavenger receptor BI, *SC4MOL* methylsterol monooxygenase 1, *SVR* sustained virologic response

### Viral hepatitis

The most common causes of viral hepatitis comprise five unrelated hepatotropic viruses, namely, hepatitis A virus (HAV), HBV, HCV, HDV, and HEV. Viral infections are initially recognized by host innate pattern recognition receptors (PRRs), including TLRs, NLRs, RIG-I-like receptors, and C-type lectins^43,44^. The early PRR-mediated innate immune response is also required for orchestrating the development and activation of adaptive immune responses, which ultimately resolve viral infections and provide long-term protection against subsequent infection. Various innate immune cells, such as natural killer cells, dendritic cells, neutrophils, and macrophages, are involved in the control of viral infection and immunopathogenesis of viral hepatitis.

As miR-223 regulates the differentiation of several key factors (e.g., neutrophils, monocytes, and granulocytes) of innate immune response, it may affect the early stage of viral infection. Studies have shown that miR-223 expression is affected by HCV infection and treatment-based viral cures. The plasma level of miR-223 is significantly increased by at least sevenfold in patients, achieving sustained virologic response (SVR), after treatment compared with the baseline level but not in patients who have relapsed. The increased miR-223 level indicates the potential antiviral functions of the miRNA in SVRs and/or a physiological response to viral clearance^45^. However, it remains unclear how high levels in the plasma relate to the expression levels of this miRNA in liver tissues. In another study, analysis of a miRNA panel in fresh liver biopsies showed that hepatic expression of miR-223 as well as other immunological and inflammatory pathway-associated miRNAs is downregulated by chronic HCV infection. Dysregulated miRNAs may be linked to chronic liver inflammation and subsequent complications by regulating the NF-κB pathway by targeting its key signaling molecules^46^. In hepatitis B patients, the results have shown that miR-223 is elevated in serum from patients with chronic hepatitis B to a similar extent as in patients with HCC, suggesting the strong potential of miR-223 to serve as a novel biomarker for liver injury but not specifically for HCC^47^. An additional study has shown that HBsAg particles circulating in HBV carriers can carry Ago2-associated protein and cellular miRNAs, especially liver-specific miRNAs and immune regulatory miRNAs (such as miR-223). These HBsAg-associated miRNAs may affect viral persistence and aid in explaining the clinical evidence that most efficient immune controls of HBV infection are associated with the loss of serum HBsAg. This finding also opens a new avenue for research to perceive the interactions between miR-223 and HBV infection^48^. Another study has shown that miR-223 levels are downregulated in the serum of HBV-positive HCC patients compared with healthy controls, and that miR-223 is downregulated in HBV X (HBx)- or HBV-transfected HepG2 and HepG2.2.15 cells, further suggesting that viral protein can regulate miR-223 expression and subsequently regulate host function by its target genes^49^.

The above observations suggest that miR-223 has roles in the disease pathology of viral hepatitis rather than serving as a biomarker alone, and that it may act as a negative regulator of the inflammatory process for hepatitis virus infection.

### Acute liver injury

Viral infections, acetaminophen (APAP) overdose, and hepatic ischemia/reperfusion (I/R)-induced acute liver injury (ALI) may progress into acute liver failure (ALF). ALF, which is characterized by fulminant hepatic necrosis and hepatitis, hepatic encephalopathy, and an elevated prothrombin time/international normalized ratio, often causes multiorgan failure and features a high mortality rate. The mechanisms that contribute to ALF can be divided into two groups as follows: direct damage from pathogens and toxic substances, and immune-mediated liver injury^50^.

Increasing evidence has indicated that miR-223 can be used as a diagnostic biomarker in ALI. Studies have shown that miR-223 expression levels are greatly upregulated in the livers of mice after I/R injury and that the hepatic miR-223 expression level is positively correlated with serum markers of ischemic injury^51^. The serum levels of miR-223 are also elevated in mouse models of I/R-induced ALI and ALF patients, as well as those in liver samples^52,53^. Another study has shown that miR-223^−/−^ mice are resistant to Fas-induced liver injury as indicated by less tissue damage and lower elevation of serum transaminases, and that miR-223 deficiency protects against ALI by targeting IGF-1R in hepatocytes^40^. Furthermore, in patients with ALI, the hepatic miR-223 expression levels in patients who show no spontaneous recovery are significantly higher than those in patients who exhibit spontaneous recovery, suggesting that hepatic expression levels of miR-223 may also be used for predicting the outcome of ALF patients^52^.

MiR-223 has also been shown to exert hepatoprotective effects on ALI by regulating immune responses. By isolating primary Kupffer cells (KCs) from concanavalin A (Con A)-stimulated mice, the upregulation of AIM2 and IL-1β has been shown to be consistent with the downregulation of miR-223 in KCs in the early stage of ALF. An in vitro experiment further confirmed that overexpression of miR-223 inhibits IL-1β expression by indirectly regulating AIM2, suggesting a possible effect of miR-223 on Con A-induced ALF by suppressing inflammasome activation and pro-inflammatory secretion of KCs^54^. In addition to its role in macrophages, miR-223 also acts as a modulator of neutrophils, which can infiltrate livers, thereby inducing liver damage^55^. MiR-223 terminates the acute neutrophilic response by targeting IKK-α expression in APAP-induced ALI^56^.

As previously described, miR-223 has a significant role in the pathophysiology of ALF by regulating hepatocyte death and immune response. However, the expression profiles and functions of miR-223 are contradictory in different ALF models. For instance, miR-223 deficiency may protect mice from FasL/CD95L-induced liver injury, but miR-223 deficiency has no effect on I/R-induced injury but may exacerbate APAP-induced injury. The precise molecular functions of miR-223 in ALF caused by different etiologies remain unclear, and further investigations are needed to develop miRNA-based therapy for ALI.

### Drug-induced liver injury

DILI is the most common cause of ALF worldwide. The incidence of DILI has continually increased and is, therefore, recognized as a major public health concern. The majority of DILI cases that lead to ALF results from the overdose of APAP, which is one of the most commonly used antipyretic and analgesic medicine. APAP-induced DILI is considered as an “intrinsic” liver toxin and is characterized by massive and coordinated hepatocyte necrosis^57^.

There is emerging evidence that miR-223 is involved in the pathophysiology of DILI. The serum level of miR-223 is elevated in DILI patients and in mouse models of DILI^57^. Studies have shown that miR-223 limits neutrophil overactivation in APAP-induced liver injury. APAP overdose can cause the release of mitochondrial DNA (mtDNA) by damaged hepatocytes, which activates neutrophils by binding to TLR9, further aggravating liver injury. In vivo and in vitro experiments have indicated that miR-223 deficiency exacerbates APAP-induced hepatic neutrophil infiltration, oxidative stress, and liver injury, as well as enhances the TLR9 ligand-mediated activation of pro-inflammatory mediators in neutrophils. Molecular mechanism studies have shown that activated TLR9 upregulates miR-223 by enhancing NF-κB binding on the miR-223 promoter but that miR-223 attenuates TLR9/NF-κB-mediated inflammation by targeting IKK-α expression. Thus, upregulation of miR-223 may have a key role in the termination of acute neutrophilic response through a mtDNA/TLR9/NF-κB/miR-223-negative feedback loop^56^.

In addition to APAP initial toxicity in hepatocytes, a sterile inflammatory response potentially involving inflammasome activation is particularly attributed to the late-stage APAP toxicity in DILI^58^. Thus, miR-223-mediated regulation of inflammasome activation may also be a potential strategy for DILI therapy.

### Alcoholic liver disease

Excessive alcohol consumption is also a major public health problem. Although mortality from alcoholic liver disease (ALD) has declined over the last few decades, it remains the most common cause of death due to alcohol. The clinical spectrum of ALD includes steatosis, steatohepatitis, liver fibrosis, cirrhosis, sepsis, and HCC. Alcohol-induced hepatotoxicity, oxidative stress, and dysregulation of immune cell functions contribute to ALD pathogenesis^59^.

As a major innate immune cell subset, neutrophils perform a crucial role in ALD pathogenesis. Alcohol can induce function alteration of neutrophils, including oxidative burst, chemotaxis, expression of β2 integrin adhesion molecule, and production of key immune mediators^54^. Recent studies have suggested that alcohol exposure modulates miRNA expression in neutrophils. MiR-223 levels are elevated in the serum and neutrophils from ethanol-fed mice. Moreover, miR-223^−/−^ mice are more susceptible to alcohol feeding-mediated induction of hepatic neutrophil infiltration and liver injury. Mechanistic studies have revealed that miR-223 directly inhibits IL-6 expression, which subsequently inhibits p47phox expression in neutrophils, thereby indicating the role of miR-223 in blocking neutrophil infiltration and reactive oxygen species (ROS) production in ALD^60^.

As inappropriate neutrophil activation and massive infiltration of neutrophils are known to contribute to ALD pathogenesis, modulation of miR-223 can be used to protect against ALD due to its regulation of neutrophil infiltration and function. Further understanding of the role of miR-223 in ALD pathogenesis may help to identify novel therapeutic targets for this disease.

### Non-alcoholic fatty liver disease

NAFLD refers to a wide spectrum of diseases that range from simple hepatic steatosis, defined as non-alcoholic fatty liver, to non-alcoholic steatohepatitis (NASH), which may progress to irreversible liver damage, such as cirrhosis, liver failure, and HCC^61^. The two-hit theory of NAFLD pathogenesis is well known. The theory states that triglycerides first accumulate in hepatocytes followed by induction of hepatic inflammation caused by inflammatory mediators^62^. Insulin resistance, intestinal microbiota, and oxidative hepatocellular injury are also implicated in NAFLD pathogenesis. Given that miRNAs are important regulators in a wide spectrum of metabolic homeostasis processes, including fatty acid metabolism, they have emerged as attractive candidate biomarkers for monitoring NAFLD progression^63^.

MiR-223 was initially reported to be myeloid cell-specific but has recently been shown to be expressed in hepatocytes. Using cellular models of NAFLD and NASH (which are induced by oleate-palmitate (non-lipotoxic) and palmitate (lipotoxic), respectively), researchers have found that both oleate-palmitate and palmitate treatment induce variations in the intracellular and extracellular miRNome in hepatocytes, especially in the cellular model of NASH. Among the massive number of deregulated miRNAs, miR-223 has been validated as one of the two most deregulated miRNAs. The deregulation of miR-223 is time-dependent in both NAFLD and NASH models. Further miRNA target expression and pathway analyses have shown that miR-223 target genes are involved in the processes associated with NAFLD and NASH onset and progression, including fatty acid metabolism and inflammation^64^. Another study using a mouse model of choline- and folate-deficient (CFD) diet-induced NAFLD showed that miR-223 is significantly increased in the livers of CFD-diet-fed mice and negatively regulates the hepatic expression of IRP1, a key regulator of intracellular iron metabolism. The high correlation of miR-223 levels with the severity of NAFLD-specific profibrogenic changes in the liver further suggest that miR-223 may be a useful biomarker to monitor the status of iron homeostasis and NAFLD progression^65^. Moreover, miR-223 affects HDL-C uptake by directly targeting and repressing SR-BI^37^. MiR-223 also indirectly promotes ATP-binding cassette transporter A1 expression by targeting Sp3, thereby enhancing cellular cholesterol efflux. Notably, miR-223 levels can be regulated by cholesterol levels. Finally, genetic ablation of miR-223 in mice results in increased HDL-C, as well as hepatic and plasma total cholesterol levels, suggesting its key role in systemic cholesterol regulation by coordinated posttranscriptional control of multiple genes in lipoprotein and cholesterol metabolism^66^.

Currently, macrophage polarization and inflammosome activation are considered to have central roles in the pathogenesis and progression of NAFLD^67,68^. As miR-223 is an important regulator in macrophage polarization by targeting Pknox1 and in activation of inflammasomes by targeting NLRP3, miR-223 may affect NAFLD progression by controlling the phenotype switch of macrophages, which is beyond its role in directly regulating fatty acid metabolism, iron homeostasis and cholesterol homeostasis. Our unpublished data verifies this assumption as miR-223 administration alleviates NASH in high-fat diet (HFD)-fed mice by suppressing hepatic NLRP3 inflammasome activation and IL-1β release.

### Cirrhosis/liver fibrosis

Liver fibrosis is a wound-healing response to injury that occurs in most chronic liver diseases. Advanced chronic fibrosis is described as cirrhosis with loss of architecture and functional failure, as well as the development of life-threatening complications. Hepatic stellate cells (HSCs) are key effector cells in fibrogenesis. Transformation of HSCs from a quiescent, fat-storing phenotype into activated, extracellular matrix (ECM)-producing myofibroblasts results in the production of transforming growth factor-β and deposition of ECM proteins, such as collagen, into the surrounding parenchyma^69^. Recently, the role of immune cells, especially subsets of macrophages, in regulating the progression or the regression of fibrosis has become a concern. Inflammatory and profibrogenic Ly6C^hi^ macrophages or restorative Ly6C^lo^ macrophages can act as the fuel or brake of liver fibrosis, respectively, indicating that macrophages act as key regulators in fibrogenesis^70^.

Considering its role in monocyte/macrophage differentiation, dysregulated miR-223 may also be implicated in the development of liver fibrosis. Several studies have shown that the serum miRNA panel, including miR-223, is deregulated in HBV/HCV-related liver fibrosis and is even correlated with the stage of liver fibrosis, indicating the possible use of circulating miR-223 for non-invasive diagnosis of liver cirrhosis^71^. The Metavir fibrosis score assay for liver histology shows that serum miR-223 is upregulated in significant fibrosis ( ≥ F2) compared with no/mild fibrosis (F0–F1). The model including miR-223 has high diagnostic accuracy for ≥ F3 and is superior to APRI and FIB-4 in discriminating ≥ F3 and F4^72^. However, in HBV-related liver fibrosis identified with the Scheuer scoring system, the serum miR-223 level is significantly downregulated as fibrosis progresses from S0–S2 (early fibrosis) to S3–S4 (late fibrosis). The model including miR-223 is associated with a high diagnostic accuracy in discriminating S0–S2 from S3–S4, and it also superior to APRI and FIB-4 for discriminating liver fibrosis progression^73^. These findings suggest that miR-223 can also be used for staging HCV- or HBV-associated liver fibrosis. In mouse models of repetitive CCl_4_ injection or bile duct ligation-induced liver fibrosis, researchers have found that hepatic miR-223 expression is significantly dysregulated upon induction of liver fibrosis and that this upregulation is restricted to hepatocytes and correlated with degree of liver injury and hepatic cell death. However, in the same study, functional experiments reveal no differences in the degree of liver fibrosis as miR-223^−/−^ mice behave identically to wild-type mice, highlighting the complex role of miR-223 in the development of liver fibrosis^52^.

Thus, in addition to be used as a novel non-invasive biomarker for diagnosis and staging liver fibrosis alone, miR-223 may have a role in regulating disease pathology. Increasing studies have shown that the progression of liver injury and fibrogenesis in various liver pathogeneses is driven by NLRP3 inflammasome activation in macrophages^74^. As miR-223 suppresses the activation of inflammasomes by targeting NLRP3, it may control the progression of liver fibrosis by modulating the phenotype and function of macrophages.

### Hepatocellular carcinoma

HCC is the third most common cause of cancer-related deaths, which may occur by chronic HBV infection, chronic HCV infection, exposure to toxins (e.g., alcohol and aflatoxin), and NAFLD. Multiple biological mechanisms, such as inflammation, oxidative stress, hypoxia, tumor microenvironment, and other molecular events, facilitate tumor initiation, progression, and metastasis. Due to their role in promoting angiogenesis, proliferation and survival of cancer cells, as well as suppression of antitumoral immune responses, tumor-associated macrophages (TAMs) may also be involved in HCC growth and metastasis^75^.

MiRNAs have crucial functions in regulating HCC tumorigenesis and metastasis signaling networks. Wong et al.^39^ revealed that miR-223 is downregulated in HCC patients irrespective of viral associations and the decrease in miR-223 may abate its suppression on cell viability by targeting STMN1. Another study has shown that miR-223 transfection specifically suppresses cell growth and promotes apoptosis in HCC cells through inhibition of the mTOR pathway by targeting Rab1^76^. Further studies have demonstrated that miR-223 is regulated by HBx and alcohol intake^51,77^. The expression of miR-223 is higher in HBV^+^ HCC patients with long-term alcohol consumption compared with HBV^+^ HCC nondrinkers or healthy controls. Knockdown of miR-223 blocks the effect of acetaldehyde by inhibiting cell growth *via* the miR-223/Fbxw7 axis^77^. In addition to its role in HCC growth, downregulation of miR-223 promotes HCC metastasis. Enhancing miR-223 expression effectively inhibits HCC metastasis by targeting integrin αV^24^. Furthermore, miR-223 modulates multidrug resistance via downregulation of ABCB1 in HCC cells^78^.

Several studies have explored the possibility of circulating miR-223 as a diagnostic tool in HCC. Comparison of the circulating levels of miR-223 in serum samples of HCC patients, chronic liver disease patients, and healthy volunteers has shown that serum miR-223 levels are significantly lower in HCC patients than in the other two groups^79^. Other studies have also shown that serum miR-223 is decreased in HCC patients and can be used as novel non-invasive biomarker of HCC alone or in a panel^73,80,81^. Moreover, several studies have indicated that serum miR-223 is also a good biomarker for predicting the outcome of Sorafenib-treated HCC or recurrence following liver transplantation^82,83^.

Increasing evidence has indicated that miR-223 is closely associated with tumor microenvironment. TAMs may transfer miR-223 to HCC cells in a contact-dependent manner involving gap junctions, thereby inhibiting HCC cell proliferation by targeting the STMN1 and IGF-1R genes^84^. TAMs also promote tumor growth and metastasis by acting as myeloid-derived suppressor cells (MDSCs) that suppress T-cell responses by secreting cytokines and growth factors. Compared with MDSCs from the spleen of disease-free mice, miR-223 expression is lower in tumor-induced MDSCs. MiR-223 inhibits the differentiation of bone marrow cells (BMCs) into MDSCs by targeting myocyte enhancer factor 2C, thereby suppressing tumor growth in mice infused with miR-223-engineered BMCs^85^. It is well known that M2 phenotype macrophages promote tumor invasiveness and contribute to poor prognosis. Thus, miR-223 may also participate in tumor development via driving TAMs to the M2 phenotype by targeting Pknox1, which is an essential regulator for macrophage polarization^41^.

Thus, miR-223 may regulate HCC tumorigenesis and metastasis by suppressing the proliferation of HCC cells directly or affecting the tumor microenvironment.

## Conclusions and prospects

Based on its role in regulating immune cell function, as well as liver physiology and pathology, miR-223 is regarded as an attractive biomarker and therapeutic target for the diagnosis and treatment of liver diseases. However, circulating and hepatic miR-223 features different expression profiles in different types of liver diseases, suggesting the necessity for further large-sample clinical studies in determination of its diagnostic value in liver pathologies. Several studies have shown the therapeutic effect of miR-223 in liver injury. For instance, bone marrow-derived mesenchymal stem cell (BMSC)-secreted miR-223-containing exosomes (BMSC-exo) prevent liver injury in an autoimmune hepatitis mouse model by suppressing hepatic NLRP3 and caspase 1, and modification of miR-223 further improves its therapeutic efficacy against autoimmune hepatitis^86^. We have also shown that miR-223 agomir administration alleviates steatohepatitis in mice with HFD-induced NASH (unpublished data). Miravirsen, a locked nucleic acid-modified DNA phosphorothioate anti-sense oligonucleotide that inhibits miR-122, and MRX34, a liposome-formulated mimic of the miR-34 tumor suppressor miRNA, have shown significant antiviral effects on chronically infected HCV patients (phase 2a trial) and antitumor activity in patients with refractory advanced solid tumors (phase 1 trial), respectively^87,88^. Given the association of dysregulated miR-223 with liver inflammation, fibrosis, steatosis, and HCC, miR-223-based therapy may provide a novel strategy in the context of advanced liver diseases and HCC. However, miR-223-based therapy still faces some challenges. First, there are divergent views on the role of miR-223 treatment in similar liver diseases. For instance, miR-223 deficiency protects against Fas-induced liver Injury, whereas upregulation of miR-223 alleviates APAP-induced liver failure. Thus, more studies are necessary to elucidate the controversial role of miR-223 in liver injury and to tailor miR-223 treatment regimens to liver injury induced by specific factors. Second, miR-223-derived drugs need to be pharmacologically targeted for cell-specific delivery of the agent. Neutrophilic miR-223 ameliorates liver injury by inhibition of neutrophil overactivation, whereas hepatic miR-223 promotes hepatocyte apoptosis. Therefore, selective and accurate delivery of miR-223 to target cells is necessary to increase the therapeutic potential and reduce possible side-effects for the treatment of liver disease. Third, the therapeutic outcomes of miR-223-based treatment are based in mouse models, and more studies with larger animals are necessary to further evaluate its effectiveness on specific liver diseases. Finally, studies should further identify the major miR-223 targets and understand the role of miR-223 in the link between inflammation and cancer, which may provide better insight into the biology of miR-223 and contribute to develop safe, accurate, and specific therapeutics for liver diseases.
